# Meteorological Factors for Dengue Fever Control and Prevention in South China

**DOI:** 10.3390/ijerph13090867

**Published:** 2016-08-31

**Authors:** Haogao Gu, Ross Ka-Kit Leung, Qinlong Jing, Wangjian Zhang, Zhicong Yang, Jiahai Lu, Yuantao Hao, Dingmei Zhang

**Affiliations:** 1 Department of Medical Statistics and Epidemiology, School of Public Health, Sun Yat-sen University, Guangzhou 510080, China; guhaogao@mail2.sysu.edu.cn (H.G.); jingqinlong@126.com (Q.J.); zhangwj35@mail2.sysu.edu.cn (W.Z.); lujiahai@mail.sysu.edu.cn (J.L.); haoyt@mail.sysu.edu.cn (Y.H.); 2 Health Information Research Center, School of Public Health, Sun Yat-sen University, Guangzhou 510080, China; 3 Guangdong Key Laboratory of Medicine, School of Public Health, Sun Yat-sen University, Guangzhou 510080, China; 4 Sun Yat-sen Global Health Institute, School of Public Health, Sun Yat-sen University, Guangzhou 510080, China; 5 Division of Public Health Laboratory Sciences, School of Public Health, The University of Hong Kong, Hong Kong Special Administrative Region, Hong Kong, China; yssun@hku.hk; 6 Stanley Ho Centre for Emerging Infectious Diseases, The Chinese University of Hong Kong, Hong Kong Special Administrative Region, Hong Kong, China; 7 Guangzhou Center for Disease Control and Prevention, Guangzhou 510440, China; yangzc@gzcdc.org.cn

**Keywords:** dengue fever, boosted regression trees, meteorological effects

## Abstract

Dengue fever (DF) is endemic in Guangzhou and has been circulating for decades, causing significant economic loss. DF prevention mainly relies on mosquito control and change in lifestyle. However, alert fatigue may partially limit the success of these countermeasures. This study investigated the delayed effect of meteorological factors, as well as the relationships between five climatic variables and the risk for DF by boosted regression trees (BRT) over the period of 2005–2011, to determine the best timing and strategy for adapting such preventive measures. The most important meteorological factor was daily average temperature. We used BRT to investigate the lagged relationship between dengue clinical burden and climatic variables, with the 58 and 62 day lag models attaining the largest area under the curve. The climatic factors presented similar patterns between these two lag models, which can be used as references for DF prevention in the early stage. Our results facilitate the development of the Mosquito Breeding Risk Index for early warning systems. The availability of meteorological data and modeling methods enables the extension of the application to other vector-borne diseases endemic in tropical and subtropical countries.

## 1. Introduction

Dengue fever (DF) is a widespread and potentially life-threatening vector-borne viral disease, which causes up to 50 to 100 million infections annually in more than 100 endemic countries, placing almost half of the world’s population at risk [1]. Some researchers even believe that the total number of suspected dengue cases might be underestimated (underreporting at the global scale), with potentially 390 million people infected with dengue worldwide [2]. Four closely-related dengue viruses [3] are transmitted by *Aedes* mosquitos, such as *Ae. aegypti* and *Ae. albopictus*. Although early detection and access to proper medical care lower the fatality rates to less than 1% [1], DF infection can develop into a more serious and complicated situation of severe dengue (previously known as dengue hemorrhagic fever and dengue shock syndrome) with a fatality rate of as high as 44% [4]. Several hundred thousand cases of severe dengue were estimated to have occurred every year since the 21st century began [5]. The understanding of the dengue spread pattern and the identification of the risk factors facilitate the development of early warning systems and timely precautions to control the DF epidemic.

Guangzhou, a subtropical megacity that is the provincial capital of Southern China, has been suffering from DF annually. *Ae. albopictus* accounts for most DF cases in Guangzhou, and although significant improvements in public health have been made by the local government over the past 30 years, geographical expansion of DF resulted in districts with an average incidence of >2.5/100,000, which increased from five (1978–1988 and 1989–1999) to seven (2000–2009) [6]. Cases are usually observed between May and November and peak between August and October, and a long-term trend with a three-year to five-year cyclical pattern (for 1978–2009) was identified [6]. The government adopted several approaches to contain the spread of DF, including regular surveillance involving weekly collection of vector data, such as the Breteau Index; dispensing the pesticide parathion to drains; public health education in communities, factories, hospitals, and schools; and research on combating dengue, such as funding agencies involved in vaccine development or related fields. Nevertheless, DF is still plaguing Guangzhou, and although the unprecedented outbreak of DF in 2014 with 37,354 laboratory-confirmed cases (47,056 cases nationwide) may be highly correlated with the unusual imported cases, effective measures for dengue control are yet to be found [7,8].

Various factors affect the incidence of DF, including the quantity of the circulating virus, the size of the susceptible population, the preventive measures against infection and transmission, and the social and environmental conditions. Vector-borne infectious diseases are strongly influenced by the combined effects of land use and extreme climatic events [9], and a considerable number of previous studies had focused on the influence of climatic factors on the prevalence of DF [10,11,12]. Particularly, extreme climatic events affect the vector more intensely than they do humans. For example, a moderate cold air outbreak may drastically reduce the vector population, but only induce minor inconvenience to humans. Dengue has mainly expanded at the global scale because of trade and globalization. Among the various causes of dengue, *Ae. albopictus* has been introduced to the USA and Europe through the trade of recycled tires and lucky bamboo plants [13].

In recent years, dengue has been expanding globally possibly because of global warming [14], and meteorological factors are key to the space–time dynamic of DF transmissions [15]. Climate affects DF infection in various ways. For example, extreme heavy rainfall may flush away mosquitoes, but residual water may become a perfect breeding site for dengue mosquitoes [16,17], and evidence that seasonal fluctuations of densities of immature *Ae. albopictus* were correlated with dengue seasonality exists [18]. Rainfall affects the frequency of the activity of mosquitos, and humans may avoid outdoor activities during rainy days which, in turn, reduces exposure. Among other environmental factors, temperature and precipitation are most cited and included in statistical models to determine and predict dengue transmission [19], and a number of recent studies have revealed that the *Aedes* vectors and dengue transmission are sensitive to daily extremes (minimum and maximum temperatures) [20,21], and the values of these extreme high and low temperatures vary by mosquito and virus [22]. Humidity and air pressure, which are considered potential predictors of DF [10], are also well-known factors in ecological studies. Previous time series analysis concerning dengue cases in 2001–2006 indicated that temperature and humidity peaks are leading dengue incidence by one month in Guangzhou [23]. However, the lag period varies by time and location, as shown in other studies [24,25].

In this study, we investigated the relative influence of various environmental factors on the daily incidence of DF in Guangzhou and the relationships between the environmental predictors and the response dengue cases. We also tested the most significant lag times of the meteorological effects. Our results showed complex effects on the risk for DF infection among different meteorological factors. These results provided additional insight into the timing and strategies for DF control and prevention in Guangzhou and in tropical and subtropical countries.

## 2. Materials and Methods

### 2.1. Ethics Statements

This study was based on official DF surveillance data in Guangzhou. Analyses were conducted at the aggregate level and no confidential information was involved. We obtained ethical approval from the Ethical Review Committee of the School of Public Health, Sun Yat-sen University (2015[024]).

### 2.2. Study Area

The study area was Guangzhou, the capital territory of Guangdong Province, which accounts for most of the DF cases in China. Guangzhou is located between 112°57′ E to 114°3′ E and 22°26′ N to 23°56′ N, with a humid subtropical hot summer climate that is mild with dry winters, hot humid summers and moderate seasonality (Köppen-Geiger classification: Cwa). The wet monsoon in Guangzhou is mainly affected by seasonal winds, which causes a hot and humid climate with an average temperature of approximately 28 °C and average precipitation between 220 mm/month and 400 mm/month in summer (June, July, and August). More monthly meteorological dynamics could be found at Guangzhou Climate & Temperature website [26]. The wet monsoon in Guangzhou is most favorable for dengue outbreaks based on the climatic relationships discussed by previous literature [21,23,24]. More than 16 million people live in this megacity, with an area of 7434 km^2^, and suffer an annual attack of DF.

### 2.3. Data Sources

#### 2.3.1. Epidemiological Data

In China, dengue is a legally-notifiable infectious disease. Suspected dengue cases are required to be confirmed by serological tests (IgM against DENV), and laboratory-confirmed cases are submitted to the Notifiable Infectious Disease Report System at the Guangzhou Center for Disease Control and Prevention (CDC). Detailed information, including the dates of onset of the laboratory-confirmed cases of DF from 1 January 2005 to 31 December 2011 (updated daily), was obtained from the Guangzhou CDC.

#### 2.3.2. Meteorological Data

Daily meteorological data comprising average relative humidity; average, minimum, and maximum temperatures; average air pressure; average precipitation; average wind speed; and sunshine duration for Guangzhou between 2005 and 2011 were obtained from the National Meteorological Information Center in China [27].

### 2.4. Statistical Analyses

#### 2.4.1. Data Preprocessing

We recorded the number of daily DF cases from 2005 to 2011. Considering that the assumptions of normality were invalid even after extreme transformation, we recoded the daily case numbers into 0 or 1 representing no case or at least one case, respectively. The corresponding daily meteorological lag data were also obtained.

#### 2.4.2. Predictor Selection

Various meteorological factors were included in the predictor pool. The linear correlations among the various predictors (e.g., variance inflation factor (VIF)) were evaluated to avoid cases of high collinearity and overfitting. After we confirmed the collinearities among parameters, we included them separately in different models. From the performance of the models (using the average area under the curve (AUC) value of 60 lag models as criteria), only unique predictors were included in the model development.

#### 2.4.3. Boosted Regression Tree Models

A boosted regression tree (BRT) model with Bernoulli distribution was used to explore the influence of climate parameters on DF. Developed by Friedman, the BRT model is different from traditional statistical or machine learning methods [28]. The BRT model combines the algorithms of regression trees that use recursive binary splits to relate a response to their predictors and boosting that built a large ensemble of small regression trees to improve predictive performance. As proposed in other studies, a learning rate of 0.01 and a tree complexity of 5 were applied in this study to ensure a sufficient number of trees for minimum error and to avoid a long and expensive calculating time. A 10-fold cross-validation was used in the BRT modeling procedure: Data are divided into 10 subsets, with stratification by prevalence. The cross-validation selection process then fits a boosted regression model of increasing complexity, calculating the residual deviance at each step along the way. After each fold was processed, the function calculates the average holdout residual deviance and its standard error and identifies the optimal number of trees as that at which the holdout deviance is minimized. In addition to quantifying the relative contributions of different meteorological factors, we were able to interpret the relationships between these continuous variables and the risk for DF using partial dependency plots, thus identifying the high-risk conditions for DF.

#### 2.4.4. Detecting Lag Time

The AUC for the training and cross validation data was used as a measure for evaluating the performance of different models with lag time of 0 day to 120 days.

## 3. Results

### 3.1. Distributions of Dengue Fever Cases

**Figure 1:**
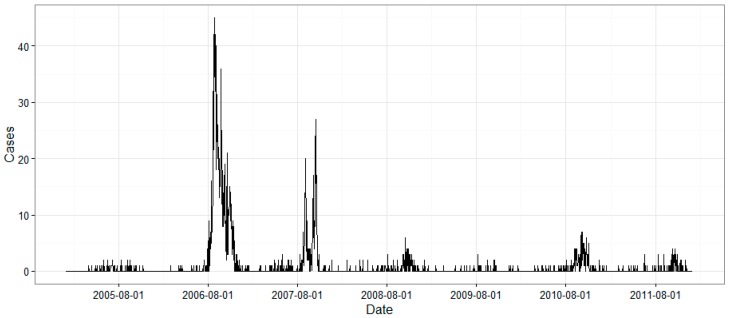
Time series of the date of onset of daily DF cases in Guangzhou from 2005 to 2011.

The number of daily DF cases in Guangzhou between 2005 and 2011, totaling 2616 cases, is shown in Figure 1. Sporadic cases were identified in the summer every year, with two peaks in 2006 and 2007, in which the number of daily cases reached more than 40.

### 3.2. The Results of Predictor Selection

All of the eight predictors were fitted into a linear regression model from which the VIF values were calculated. The results showed that four predictors, that is, daily average air pressure, average temperature, maximum temperature, and minimum temperature, exhibit significant collinearity (VIF values > 4, i.e., 4.88, 143.04, 66.62, and 32.66, respectively). These four predictors were included in the BRT model separately, and the models with average temperature attained the highest average AUC (for the training data) value of 60 lag models (0.8171 vs. 0.8043, 0.8096, and 0.8148 for air pressure, maximum temperature, and minimum temperature, respectively). Thus, the daily average accompanied by other climatic factors was selected for the subsequent analyses.

### 3.3. The Lag Time of Dengue Fever in Guangzhou

**Figure 2:**
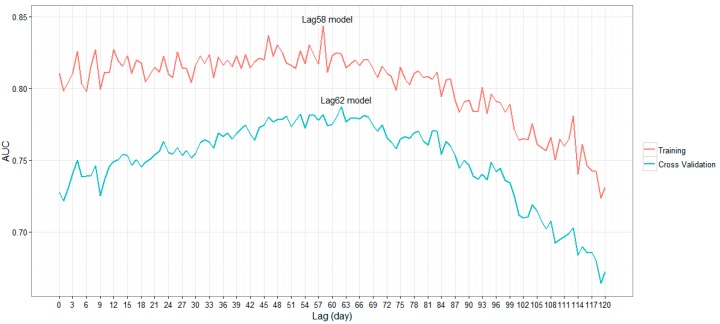
The AUC values for 121 BRT models.

**Figure A1:**
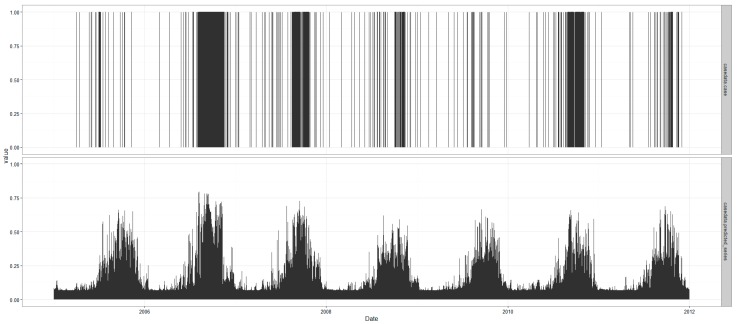
The predicted values and the actual case series.

The AUC values of 120 lag models, alongside the model with no lag time, are plotted in Figure 2. Two high local AUC peaks for the 58 and 62 lag days could be identified. The AUC values for the training and cross-validation data increased with the increase in lag time from 1 day to approximately 60 days and decreased gradually afterward. The dynamics of different lag models and the two specific lag models that attained the highest AUC among the two datasets were analyzed in more detail. A comparison between the predicted values (lag-62 model) and the actual case series (in the absence/presence data) is presented in Appendix Figure A1.

#### The Relative Contribution of Different Meteorological Factors

Table 1 shows that the rankings of the relative contributions of different factors for the two selected models were identical. The same ranking was also valid for the average of the relative contributions of 121 models, accompanied by the ranking of the range, indicating that the factors were stable among different models. In addition to the average, the standard deviation and range of all of the 31 models were calculated. Although the standard deviation of temperature was relatively high, it was the most important factor among the models.

Daily average temperature was identified to be the most important factor, accounting for more than 28%, and up to 62%, of the relative contribution, followed by humidity that accounted for approximately 18% (13.18% to 27.09%). Precipitation and sunshine duration affected DF slightly lesser than humidity (approximately 14% and 11%, respectively), and wind speed was the factor with the least contribution (approximately 8%).

**Figure 3:**
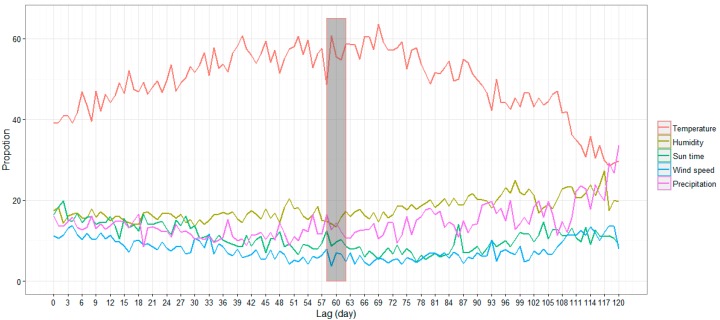
The dynamics of the relative contributions of the variables for 121 BRT models (the gray rectangle indicates the period of the optimal lag time, i.e., 58 lag days to 62 lag days).

The dynamics of the relative contributions of different variables (Figure 3) showed that the relative contribution of the daily average temperature increased unsteadily from the lag time of 0 day to approximately 60 days. The relative contribution of daily average humidity remained stable most of the time, whereas the relative contributions of the other variables, including precipitation, sunshine duration, and wind speed, decreased to different levels with the increase in lag time from 0 day to 60 days.

### 3.4. The Relationships between Meteorological Factors and the Risk for Dengue Fever

**Figure 4:**
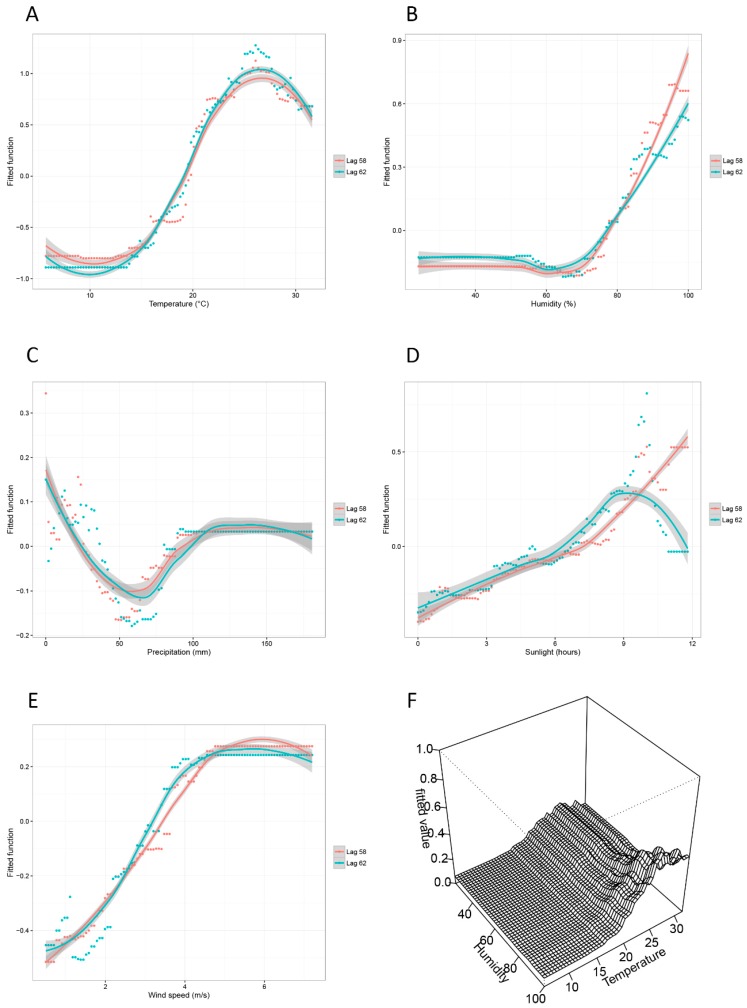
The partial dependency plots for five meteorological factors and the joint partial dependency plot for daily average temperature and daily average humidity. (**A**) The relationship between daily average temperature and the risk for DF epidemic; (**B**) The relationship between daily average relative humidity and the risk for DF epidemic; (**C**) The relationship between daily precipitation and the risk for DF epidemic; (**D**) The relationship between daily sunshine duration and the risk for DF epidemic; (**E**) The relationship between daily average wind speed and the risk for DF epidemic; (**F**) The interaction between daily average temperature and daily average relative humidity.

Daily average temperature contributed most strongly to the risk for DF in Guangzhou. Two positive relationships with temperature were observed in the lag-58 and lag-62 models (Figure 4A). The risk for DF increased steeply as the daily average temperature increased from 13 °C to 28 °C. However, the risk values in the lag-62 model seem to be more sensitive to high temperature, that is, a noticeable difference for the lag-62 model having higher risk values can be observed when temperature ranged from 20 °C to 30 °C.

Significant positive relationships between DF infection and humidity were observed in the lag-58 and lag-62 models (Figure 4B). Daily precipitation could also have a complex relationship with the risk for DF (Figure 4C). In the absence of precipitation, significantly high risks for DF were observed in both models. However, as precipitation increased from 0 mm to 100 mm, the risk values initially decreased at approximately 60 mm/day and then increased. The risk for DF was highest in conditions with relatively low (i.e., less than 30 mm) and relatively high (i.e., greater than 100 mm) precipitation.

Daily sunshine duration exerted a similar influence on the risk for DF between the two lag models when the sunshine durations were less than 9 h (Figure 4D). However, the situation became complicated when the sunshine duration was more than 10 h; the risk values of the lag-58 model initially decreased and then rapidly increased to a peak, whereas the risk values of the lag-62 model decreased steeply with the increase in sunshine duration.

The risk increased with the increase in daily average wind speed from 0 m/s to 4 m/s for the lag-58 or lag-62 model, but with a faster pace for the lag-62 model (Figure 4E).

A joint partial dependency plot was presented to view the interactions between daily average temperature and humidity directly. As shown in Figure 4F, the condition with high temperature (20 °C to 30 °C) and high humidity (90% to 100%) in the lag-62 model was considered to be at high risk for the DF epidemic.

## 4. Discussion

Guangzhou is a large, densely populated city in China that suffered most seriously from DF during the past decade. From the laboratory-confirmed cases of DF from 2005 to 2011, two major outbreaks in 2006 and 2007 were identified. The cases in these two years accounted for more than 80.7% of the cases in the entire study period. Environmental variables usually co-vary, leading to difficulties in drawing definite conclusions about the effect of any specific variable. For instance, as the climate in Guangzhou is distinct from season to season, temperature, precipitation, and sunshine duration reach their peaks in the summer every year. Although high temperature may be associated with more dengue cases, whether it is a causative factor for DF remains uncertain. However, we are able to obtain more detailed information regarding the effects of all variables by adapting sufficient data in the BRT model.

The average AUC values of the 121 models (0.802 and 0.749 for training and cross-validation, respectively) indicate that the meteorological factors in Guangzhou can fit their associated onset situation of DF well. The performance of the models increased gradually and peaked at the time lag of approximately 60 days, indicating that two months may be the optimal time for forecasting DF epidemics using the BRT models. Considering that 60 days is significantly longer than the full vector life cycle, the viral transmission period, and the incubation time of DF, we believe that the meteorological factors and the DF epidemic may have some accumulative affects.

The two representative lag models were selected based on their fitting performance (AUC values) in two different datasets, namely, the training data and the 10-fold cross-validation data. Many aspects of these two models were similar, including the ranking of the relative contributions and the partial dependencies of different variables.

The relative influence of daily average temperature factored highly in our analysis and many other related studies, supporting the assumption that dengue mosquitoes feed more often and develop faster from larva to adult at high temperature [14,20,29]. Although relative humidity, sunshine duration, precipitation, and wind speed accounted for the low relative importance of the meteorological factors, the influence of environmental factors on DF infection is relatively complex, with different factors interacting with each other in a variety of direct or indirect ways. These less important factors should also be considered in the management of the disease.

The influences of temperature on the two models are approximately the same. The risk for DF increased with the increase in temperature. Many studies have shown that high temperature can affect the mating behaviors and reproduction activities of mosquitoes. Watts et al. proposed that increasing temperature causes more rapid viral replication and longer mosquito survival durations [30], whereas Su et al. reported that the maximum mating rates of mosquitoes at 20 °C and 25 °C were higher than that at 30 °C [31]. As the risk for dengue exceeded its average when temperature was higher than 21 °C to 22 °C, preventive measures should be taken to hinder mosquitoes from mating and reproducing, for example, dispensing pesticide or eliminating stagnant water [32].

The survival of mosquito eggs and adults can be affected by relative humidity. Newly-laid eggs are sensitive to desiccation, and moisture-related reductions in the survival of adult mosquito occur [33]. The results of our study also indicated that we should be alerted of the risk for DF as the relative humidity increases.

The relative contributions of precipitation and daily sunshine durations notably competed in our study. Figure 2 shows that the relative contribution of sunshine duration was higher than that of precipitation when the lag time is short (less than 30 days). However, when the lag time exceeded 30 days, precipitation became more important in model building. This finding makes sense when considering the different effects of precipitation and sunshine duration on DF transmission; precipitation affects DF transmission in a more prolonged manner by providing breeding sites for vectors, whereas sunshine duration more likely affects the behavior of the vectors directly.

Although extreme heavy rainfall may flush away the dengue mosquitoes [33], residual water may provide essential breeding habitats for larva and adult mosquitoes [16,17,34]. According to the definition of the China Meteorological Administration, rainstorm refers to a daily precipitation between 50.0 mm and 99.9 mm, whereas heavy rainstorm refers to a daily precipitation between 100.0 mm and 249.9 mm. Our results indicated that mild rain (0 mm to 30 mm) may facilitate the reproduction of mosquitoes. When rainstorms occur, the conditions become unfavorable for mating and hunting. However, when heavy rainstorms occur, the risks increased significantly. Therefore, a possible prevention control measure can be eliminating stagnant water after mild rain (0 mm to 30 mm) or heavy rainstorm (more than 100 mm).

Generally, the daily sunshine duration in summer in Guangzhou is more than 7 h. Sunshine duration can represent the transition from spring to summer. The majority of the cases occurred in summer, which explains the increasing risk for DF with the increase in sunshine duration from 0 h to 9 h. Specifically, when sunshine duration was approximately more than 9.5 h, the risk of DF decreased dramatically. The significant decrease in risk for DF could indicate that this sunshine duration provides good conditions for mosquito blood feeding. For example, early dusk may provide mosquitoes a longer time for hunting. Nevertheless, we assume that the sunshine duration has only a slight direct effect on DF transmission.

Wind speed was predicted to play the least important role in DF infection from 2005 to 2011. Wind speed did not exhibit consistently significant relationships with indigenous dengue in Taiwan [11]. In our study, the contribution of wind speed was relatively low. Wind velocity may be collinear with humidity or precipitation as stormy days always bring rain and wind together. As a result, the risk for DF increased steadily with the increase in wind speed from 0 m/s to 6 m/s.

There were clear variations in different optimal lags for different parameters shown in Figure 3. We believe that different meteorological factors may play more important roles at different lags, and the relationships between different lags and different meteorological parameters are worth exploring. In this study, we mainly focus on the question of how the meteorological factors in a single day can affect the incidence of DF cases. A follow up study focusing on different lags for different meteorological parameters is needed.

From the lag models, a risk estimating system could be further developed. A Mosquito Breeding Risk Index for DF can be generated by the optimal lag model; when this index is high, timely actions should be taken to eliminate the suitable breeding sites for mosquitoes. Daily, even hourly, meteorological data can be easily obtained from weather stations, allowing managers and researchers to develop lag models and risk indices, which are helpful in dengue containment and epidemic preparedness. Other factors can also contribute to DF infection, and the latent infection and mild cases of DF are limiting our ability to assess the definite infection status among the entire population. Although the current model provides a binary prediction for dengue fever risk, thus it is hard to distinguish between “significant outbreaks” and “random noise”, we could address this by incorporating more continuous case data, and applying different statistical distribution model in the following study and it would be of great help when making wise (economical) decisions. Alongside the meteorological risk factors used in this study, the exploration of relevant environmental factors and the inclusion of more high-quality case data shall facilitate the development of accurate predictive models, paving the way for an early warning system.

## 5. Conclusions

Dengue, a rapidly-spreading mosquito-borne disease, causes acute febrile illness and significant economic loss in the world. In this study, we evaluated probable lag periods and the relationships between different meteorological factors and the risk for DF in Guangzhou, a large city in China with a humid subtropical climate, inhabited by more than 10 million citizens. We analyzed seven years of daily dengue cases by BRT and identified daily average temperature as the most influential factor. Two lag periods, that is, 58 and 62 days, with the strongest meteorological signals for prediction were presented. The climatic factors presented similar patterns between these two lag models, which can be used as references for DF prevention in the early stage. For example, priority should be given to the eradication of mosquitoes (and their eggs) when daily average temperature is relatively high (more than 20 °C), when relative humidity is high (more than 90%), and in days with light rain (precipitation of 0 mm to 30 mm), long sunshine duration (more than 9 h), or high wind speed (0 m/s to 2 m/s). Our results assist in understanding the meteorological risk for DF and its lag effect, which facilitates dengue containment and epidemic preparedness. The methodology and knowledge can also inspire research on other vector-borne diseases not limited to DF in tropical and subtropical countries.

## Figures and Tables

**Table 1:** The relative contributions (%) of the variables for two selected lag models and the range and average values of all of the 121 BRT models.

Variables	Lag-58 (%)	Lag-62 (%)	Average (of 121 Models) (%)	Range (of 121 Models) (%)	Standard Deviation (of 121 Models)
Temperature	48.57	58.65	49.35	28.56 to 62.03	7.86
Humidity	14.76	17.31	17.82	13.18 to 27.09	2.75
Precipitation	16.44	10.61	14.39	8.62 to 32.32	3.95
Sunshine duration	12.33	8.69	10.64	4.81 to 19.83	3.22
Wind speed	7.89	4.74	7.80	3.3 to 13.37	2.4

## Abbreviations

The following abbreviations are used in this manuscript:

DF	Dengue fever
BRT	Boosted regression trees
AUC	Area under the curve
CDC	Center for Disease Control and Prevention
